# Adhesion molecule periplakin is involved in cellular movement and attachment in pharyngeal squamous cancer cells

**DOI:** 10.1186/1471-2121-12-41

**Published:** 2011-09-27

**Authors:** Yurie Tonoike, Kazuyuki Matsushita, Takeshi Tomonaga, Koji Katada, Nobuko Tanaka, Hideaki Shimada, Yukio Nakatani, Yoshitaka Okamoto, Fumio Nomura

**Affiliations:** 1 Department of Otorhinolaryngology, Chiba University Hospital, 1-8-1 Inohana, Chiba City, Chiba 260-8670, Japan; 2 Department of Molecular Diagnosis, Graduate School of Medicine, Chiba University Hospital, 1-8-1 Inohana, Chiba City, Chiba 260-8670, Japan; 3 Department of Clinical Proteomics Research Center, Chiba University Hospital, 1-8-1 Inohana, Chiba City, Chiba 260-8670, Japan; 4 Proteome Research Center, Proteome Research Project, National Institute of Biomedical Innovation, 7-6-8 Saito-Asagi, Ibaraki City, Osaka 567-0085, Japan; 5 Department of General and Gastrointestinal Surgery, Toho University Omori Medical Center, 6-11-1 Ohta-ku, Ohmori-nishi, Tokyo 143-8541, Japan; 6 Department of Diagnostic Pathology, Graduate School of Medicine, Chiba University, 1-8-1 Inohana, Chiba City, Chiba 260-8670, Japan

## Abstract

**Background:**

We previously reported that periplakin (PPL) is downregulated in human esophageal cancer tissues compared to the adjacent non-cancer epithelium. Thus PPL could be a useful marker for detection of early esophageal cancer and evaluation of tumor progression, but largely remains unknown in this field. To investigate PPL involvement in carcinogenesis, tumor progression, cellular movement or attachment activity, siRNAs against PPL were transfected into pharyngeal squamous cancer cell lines and their effects on cellular behaviours were examined.

**Results:**

PPL knockdown appeared to decrease tumor cell growth together with G2/M phase accumulation in cells attached to a culture dish. However, the extent of cell growth suppression, evaluated by the number of cells attached to the culture dish, was too distinctive to be explained only by cell cycle delay. Importantly, PPL knockdown suppressed cellular movement and attachment to the culture dish accompanied by decreased pAktSer473 phosphorylation. Additionally, LY294002, a PI3K inhibitor that dephosphorylates pAktSer473, significantly suppressed D562 cell migration. Thus PPL potentially engages in cellular movement al least partly via the PI3K/Akt axis.

**Conclusions:**

PPL knockdown is related to reduced cellular movement and attachment activity in association with PI3K/Akt axis suppression, rather than malignant progression in pharyngeal cancer cells.

## Background

Pharyngeal and esophageal cancers are some of the most malignant gastrointestinal tumors [1]. We previously reported that a cell adhesion molecule, periplakin (PPL), is significantly downregulated in human esophageal cancers. Immunohistochemical staining has also revealed that PPL changes its cellular localization as well as its expression levels with cancer progression [2]. In addition, PPL expression has been associated with nodal metastasis [3]. These findings indicate that PPL plays an important role in the development of esophageal and pharyngeal squamous cell carcinoma; however, the precise mechanism remains largely unknown.

PPL is a member of the plakin family comprising desmoplakin, envoplakin, plectin, and bullous pemphigoid antigen 1, which have various functions in connecting cytoskeleton elements to form intercellular junction complexes [4]. Plakin families also function as "molecular bridges" of cells that link the intracellular cytoskeleton and cell-cell junctions. For example, plectin, one of the most well-studied plakins, directly interacts with signal transductions [5,6]. PPL also plays a role as a localization signal in oncogenic serine/threonine protein kinase Akt/protein kinase B (PKB)-mediated signaling in human cancer cell lines [7]. Here, Akt/PKB is a downstream effector of phosphatidylinositol 3' kinase (PI3K) and functions as a critical regulator of cell survival and proliferation. Furthermore, activation of the PI3K/Akt/PKB pathway is emerging as a central feature of epithelial-mesenchymal transition (EMT), which is believed to be a crucial event in tumor development [8].

In this study, we examined changes in cell growth, morphology, migration, and attachment activity following siRNA against PPL to investigate the involvement of PPL in tumor development. We found that PPL knockdown was related to a reduction in cellular movement and attachment activity, and was accompanied by PI3K/Akt axis suppression in pharyngeal cancer cells; it was potentially related to EMT promotion.

## Results

### Knockdown of PPL expression reduced the number of cells attached to the culture dish

**Figure 1:**
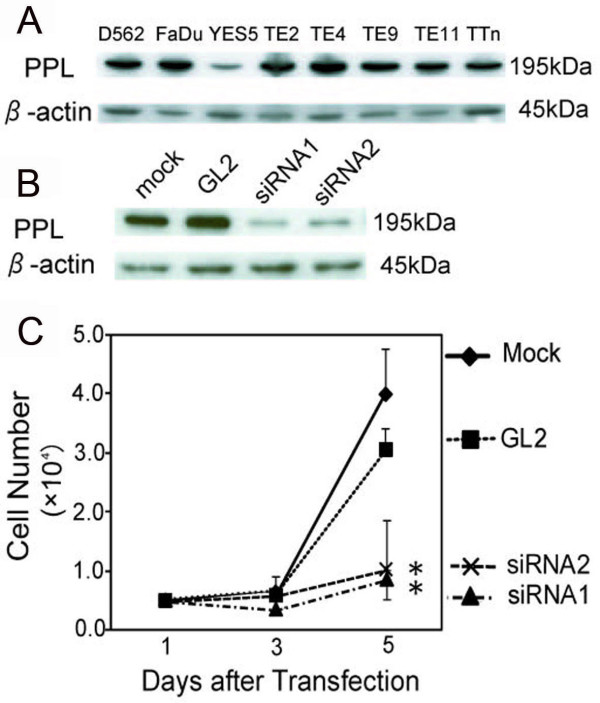
**Downregulation of PPL by siRNA decreased the number of attached D562 cells**. (A) PPL expression was detected in human pharyngeal (D562 and FaDu) or esophageal (YES5, TE2, TE4, TE9, TE11, and T.Tn) cell lines by western blotting. (B) siRNA1 and siRNA2 against PPL was shown in D562 cells. Total cell extracts were collected 72 h after siRNA treatment, and 5 μg protein aliquots were blotted with antibodies against the indicated proteins. siRNA (GL2) against firefly luciferase was used as a control. β-actin was the internal control. (C) Downregulation of PPL inhibited cell growth and decreased the number of attached cells. The number of viable cells was measured at different times by trypan blue exclusion. Values represent the mean ± S.E. from 3 separate experiments. Asterisks (*) indicate a p value less than 0.01 based on a t-test using Day 5 data.

Since PPL expression is decreased in esophageal carcinoma tissues [2], we tried to determine the effects of PPL downregulation on cancer cell activities. First, endogenous PPL expression in hypopharyngeal squamous carcinoma (D562 and FaDu) and esophageal squamous carcinoma (YES5, TE2, TE4, TE9, TE11, and TTn) cells was examined (Figure 1A). D562 cells were selected because siRNA against PPL was most efficient among these cell lines (Figure 1B, data not shown). In D562 cells, PPL siRNA inhibited cell growth and reduced the number of cells attached to the culture dish (Figure 1C).

### Knockdown of PPL expression increased the proportion of G2/M phase cells attached to the culture dish

**Figure 2:**
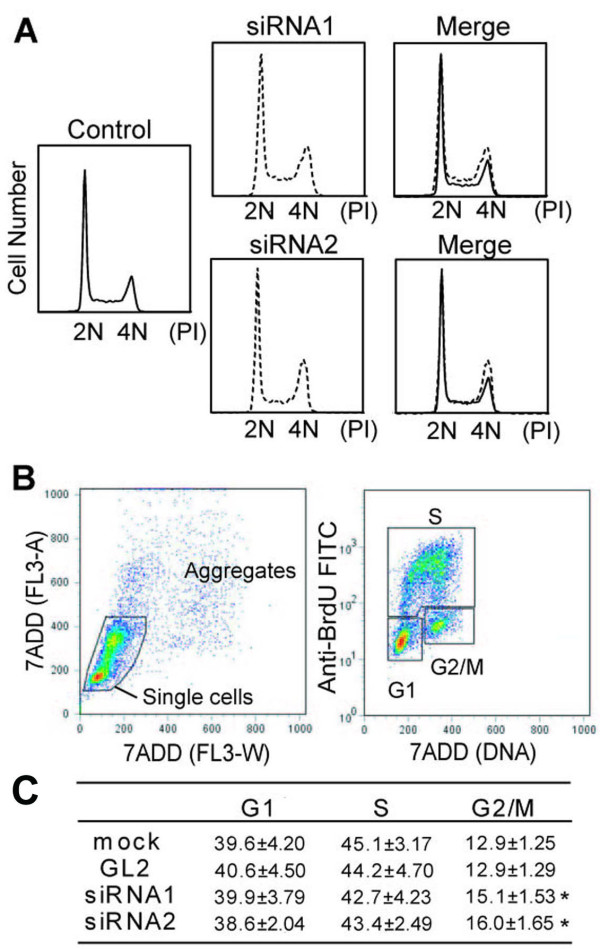
**siRNA against PPL delayed the cell cycle at the G2/M phase**. D562 cells were transfected with control siRNA or siRNA 1 against PPL. (A) Downregulation of PPL delayed cell cycle progression during the S and G2/M phases. Cells were harvested after 72 h, fixed, and processed as described in "Materials and Methods." DNA content was measured by flow cytometry after propidium iodide staining. (B) (C) Flow cytometry analysis of cell cycle parameters was performed after double staining for BrdU incorporation (S phase cells) and 7-AAD (DNA content). To measure direct cell cycle distributions in cell cycle phases, exponentially proliferating cells were labeled with 10 μM BrdU for 1 h and processed for 2-color FACS, as described in "Materials and Methods." Values represent the mean ± S.E. from 5 separate experiments. Asterisks (*) indicate a p value less than 0.05 based on Student's t-test.

So why was the number of attached cells to the culture dish reduced after PPL downregulation? To determine the mechanism behind this phenomenon, we first analyzed the cell cycle populations after PPL siRNAs were transfected into D562 cells. Compared to control (GL2) siRNA, cultures treated with PPL siRNA had a decreased G1 (2N) and an increased late S and G2/M cell population (Figure 2A). To verify this result, we further developed a double channel, BrdU and 7-amino-actinomycin D (7-AAD), flow cytometry protocol. By plotting BrdU incorporation (S phase cells) on the y-axis and 7-AAD (DNA content) on the x-axis, the cells could be divided into 3 populations: G1, S, and G2/M (Figure 2B, right). Thus, knockdown of PPL reduced the S population and increased the G2/M population (Figure 2C). In particular, the G2/M population increased 12.9% in the control cell culture and 15.6% in the PPL siRNA cell culture, a 20.9% increase (average of siRNA1 and siRNA2). These data suggest that PPL siRNA decreased cellular proliferation of D562 cells, at least partly by delay in the G2/M phase of cells attached to the culture dish. However, to determine the effect of PPL knockdown on cell growth more precisely, cells detached from the culture dish will need to be examined along with cells attached to the culture dish.

### Knockdown of PPL reduced cell adhesion activity

**Figure 3:**
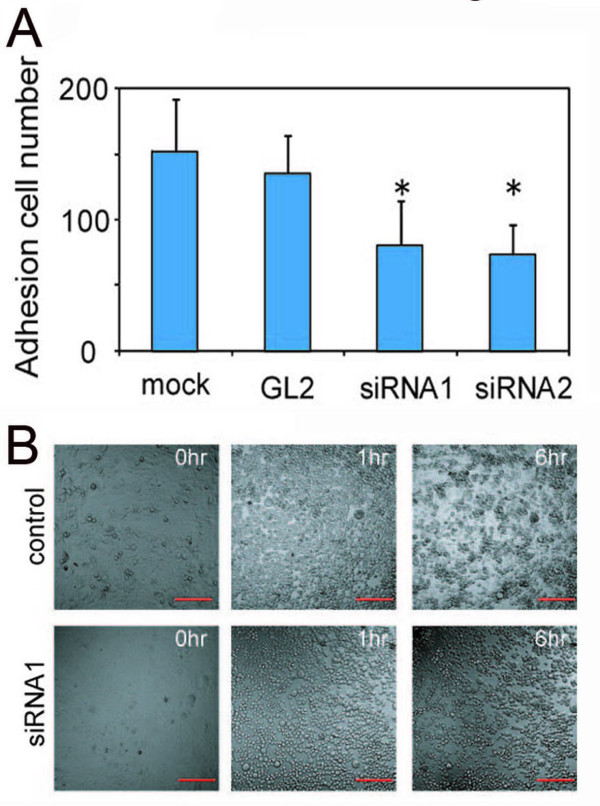
**siRNA against PPL reduces adhesion to ECM in D562 cells**. D562 cells were transfected with either control or PPL siRNA. (A) Effect of downregulation of PPL by siRNA on the adhesive characteristics of D562 cells. After 48 h, cells were trypsinized and 2 × 10^4 ^cells were resuspended in a 24-well plate coated with 200 μg of Matrigel. The plates were incubated at 37°C and adherent cells were counted. Four random fields were counted in 2 separate wells and the results were averaged. Values represent the mean ± S.E. from 3 separate experiments. Asterisks indicate a p value less than 0.05 based on Student's t-test. (B) Snapshot from a time-lapse recording of 2 transfected wells. D562 cells were transfected with either control siRNA (Additional file 1) or PPL siRNA (Additional file 2). Cells were grown to confluence in glass-bottomed dishes and EDTA (2.5 mM final concentration) was added. Changes in the morphology of the culture were recorded by live imaging. Time-lapse recordings were made every 5 min for 6 h. Bar: 200 μm.

Although siRNA against PPL increased the G2/M phase population, the decrease in the number of cells grown on the culture dish was more remarkable (Figure 1C). Nonetheless, PPL siRNA did not increase apoptosis when this was assessed by an Apopercentage assay™ (Biocolor Ltd., Newtownabbey, Northern Ireland, UK; data not shown). How then should we explain the reduction in the number of attached cells? Possible explanations include cell growth suppression, cell cycle retardation, or detachment from the culture dish. Therefore, we next examined the effects of PPL downregulation on adhesion to the extracellular matrix (ECM) by an adhesion assay. Cells transfected with PPL siRNA decreased their attachment to ECM compared with controls (Figure 3A). In order to confirm this result, we performed an ethylenediaminetetraacetic acid (EDTA) assay in which EDTA was used to gently block adhesion of D562 cells to ECM during a 1-h incubation at 37°C. Interestingly, PPL siRNA-treated and control cells lost cell-cell and cell-substrate adhesion and changed from elongated to spherical shape (Figure 3B). However, 6 h after the release of EDTA, control cells recovered their ability to adhere to ECM and began to reproliferate (Additional file 1). In contrast, PPL siRNA-treated cells remained spherical and did not proliferate, suggesting that PPL siRNA significantly attenuated cellular adhesion to ECM (Additional file 2). These data suggest that knockdown of PPL decreases cellular adhesion to ECM in D562 cells.

### Knockdown of PPL reduces cell migration activity

**Figure 4:**
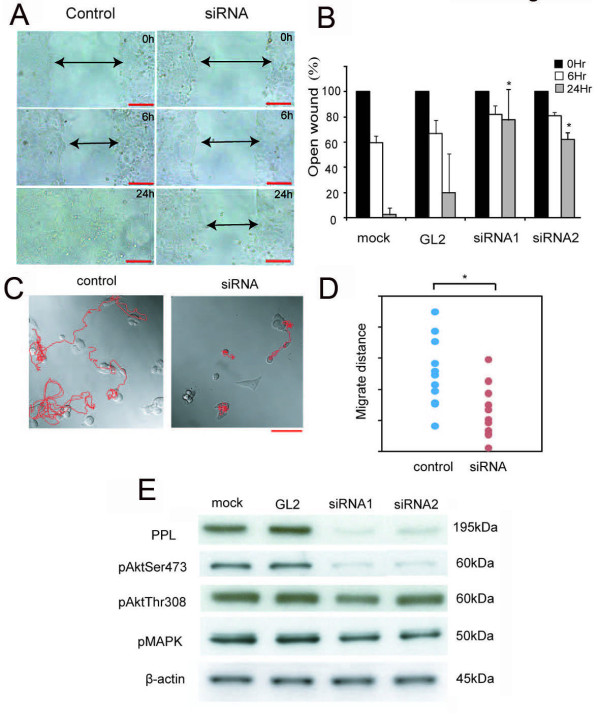
**siRNA against PPL reduces cell migration in D562 cells**. D562 cells were transfected with control and PPL siRNA. (A), (B) Quantification of wound closure in control and PPL siRNA. Each cell was grown to confluence and then wounded by scraping. For each wound, the width of the wound at 0 h was designated as 100%, and the subsequent timepoints in the graph show the relative width of the open wound. Values represent the mean ± S.E. from 3 separate experiments. Asterisks indicate a p value less than 0.01 based on Student's t-test. Bar: 200 μm. (C), (D) Paths of 2 transfected cells from a time-lapse recording. D562 cells were transfected with either control or PPL siRNA. After 24 h, cells were seeded on glass-bottomed dishes and incubated for another 24 h. Time-lapse recordings were made every 5 min for 24 h. Migration distance was measured using 10 control and 10 PPL siRNA-transfected cells. Bar: 100 μm. Asterisks indicate a p value less than 0.05 based on Student's t-test. **(**E) Downregulation of PPL resulted in reduced pAkt expression. The expression of PPL and β-actin was analyzed by western blotting with total cell extracts from transient transfections with control siRNA (GL2) and PPL siRNA 1 and 2.

Cell migration is essential for the invasion and metastasis of cancer cells. Thus, to evaluate the effects of PPL downregulation, a wound-healing assay was also performed using D562 cells. PPL siRNA-treated cells showed a remarkable delay in wound closure compared with control cells (Figure 4A and 4B). This delay in wound closure could be caused by disturbance of cellular proliferation or migration. To discriminate between these possibilities, we used time-lapse and video microscopy after transfection of PPL siRNA to compare and record cellular movement and migration (Figure 4C and 4D). As expected, PPL siRNA-treated cells showed a significant decrease in motility and a shorter tracking migration-length than controls (Additional file 3 and Additional file 4). Moreover, we injected YES5 cells (low PPL expression) and D562 cells (high PPL expression) beneath the right thigh of nude mice and monitored subsequent tumor growth. No tumor growth was observed in YES5 mice, while some tumors were observed in D562 mice (Additional file 5). These results indicate that YES5 could not migrate into mice tissues owing to its slow proliferation or weak adhesiveness to ECM.

### Knockdown of PPL decreases cellular migration via the PI3K/Akt axis

Finally, we examined changes in the expression of phospholipid signaling proteins that regulate cell migration because PPL has been reported to play a role as a scaffold in Akt/PKB signaling in human cancer cell lines [7]. The mitogen-activating protein kinase (MAPK) pathway was also examined because PI3K/Akt/PKB and the pathway are activated by G protein-coupled receptors (GPCRs) that induce signaling events [9-11], and PPL can also interact with a wide range of GPCRs and modulate their functions [12-14]. As expected, protein expression of pAktSer473 was drastically downregulated by PPL siRNA (Figure 4E). PPL also regulates keratin organization and epithelial migration, participating in the accumulation of keratin filaments to form thick cables [15,16]. In our study, PPL siRNA reduced not only the migration of assembled cells but also that of single D562 cells with pAktSer473 suppression. These data indicate that knockdown of PPL significantly decreases cellular migration in D562 cells, at least partly via the PI3K/Akt axis. Recent reports have indicated that the PI3K/Akt axis is a central feature of EMT and that Akt-induced EMT involves downregulation of E-cadherin [8,17]. As stated above, our results also showed that siRNA against PPL suppressed pAktSer473 and reduced cellular attachment activity.

### PPL expression was different among normal, atypical epithelium and cancer of the pharyngeal mucosa

**Figure 5:**
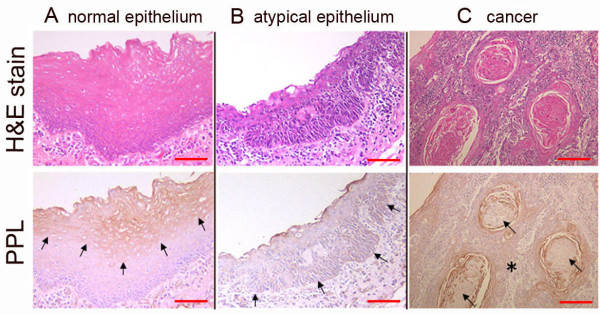
**Expression of PPL in normal, atypical epithelium and cancer of the pharyngeal mucosa**. (A) In normal hypopharyngeal mucosa, PPL is expressed in the upper layer of the squamous epithelium (arrows). (B) In atypical hypopharyngeal epithelium, PPL expression is rare (arrows). (C) In the pharyngeal cancer epithelium, PPL expression is slight and occurs on the ridge of the cornified epithelium. Note that in both the non-keratinized (asterisk) and keratinized cancer areas (arrows), PPL expression was scarce. Bar: 100 μm.

If PPL expression relates to cellular migration, movement or invasion, PPL expression should be different among normal, atypical epithelium and cancers. Under this hypothesis, we immunohistochemically examined PPL expression in excised human normal, atypical epithelium and in cancer of the pharyngeal mucosa (Figure 5). Interestingly, PPL was expressed in the upper layer of the squamous epithelium in normal hypopharyngeal mucosa (Figure 5A, arrows), whereas PPL expression was rare in atypical hypopharyngeal epithelium (Figure 5B, arrows). Note that in both the non-keratinized (Figure 5C, asterisk) and keratinized cancer areas (Figure 5C, arrows), PPL expression was scarce. Further experiments are needed to determine how PPL relates to EMT, including investigations of whether PPL knockdown affects the expression of mesenchymal markers, such as E-cahderin, fibronectin and/or N-cadherin.

## Discussion

In this study, we revealed that siRNA against PPL significantly reduced the cellular motility and adhesion activity. In addition, PPL downregulation significantly decreased cellular migration, possibly because of PI3K/Akt axis interference supported by a reduction in phosphorylated Akt at Ser473 (pAktSer473). Collectively, PPL suppression reduced cell motility, and ECM invasion, all of which generate tumor progression by increasing infiltrating activity in pharyngeal squamous cancer cells.

If PPL knockdown reduces cell adhesion activity, PPL may be associated with proliferation and/or cell death because homeostasis in epithelial cells is enforced by their structural characteristics [18,19]. Furthermore, distortion of these balances by PPL siRNA, which interferes with cell adhesion to ECM, is often associated with epithelial tumorigenesis [20]. At this point, attachment to ECM is essential to support cellular functions, including differentiation, apoptosis, proliferation, and migration.

In cancers, the hallmarks of EMT are the acquisition of a migratory and invasive phenotype and/or anoikis resistance, that is, accommodations for survival even in the absence of adhesion to ECM [21,22]. In fibroblast cells, Akt signaling enhances activation of various small guanosine triphosphatases (GTPases), resulting in actin cytoskeleton remodeling and enhanced cell motility [23,24]. In this context, the dephosphorylation of Akt and pAktSer473 induced by PPL siRNA (Figure 4E) is enlightening. LY294002, a PI3K inhibitor, suppressed cellular migration activity in D562 cell (Additional file 5). Since PI3K/Akt/mTOR pathway inhibition is reported to decrease invasion and migration of ovarian carcinoma cell lines [25,26], thus PPL siRNA potentially decreased cellular attachment, migration or movement at least partly via the PI3K/Akt axis.

Moreover, the effects of the matrix are primarily mediated by integrins, a family of cell surface receptors that attach cells to ECM and mediate mechanical and chemical signals from ECM [27]. The PI3K/Akt axis is positively regulated by integrins. Although further studies are necessary, the relationship among PPL, integrins, and the integrin-plectin-vimentin complex potentially bridges ECM and intracellular cytoskeletons [17,28,29]. To investigate the integrins involved in this impaired adhesiveness, a cell adhesion assay using other substrates such as Poly-D/L-lysin, fibronectin, or type-I collagen may be informative. For instance, apoptosis is induced when cells are detached from the surrounding ECM (anoikis) in normal cells [30]. However, cancer or metastatic tumor cells can escape anoikis and thus invade organs (anoikis resistance) [31]. Our results indicate that PPL downregulation is accompanied by PI3K/Akt axis interference which indicates EMT in pharyngeal cancers. In summary, the function of PPL relates to cellular proliferation, cell cycle regulation, cellular movement, and attachment to ECM at least partly via PI3K/Akt axis.

Thus, knockdown of PPL reduced ECM attachment and cellular proliferation. Further studies are required to investigate the role of PPL as a candidate biomarker for diagnosis and treatment of pharyngeal cancers.

## Conclusion

The down-regulation of PPL significantly reduced cellular motility and adhesion activity along with phosphorylated AktSer473 suppression. Thus, PPL was positively involved in cellular movement and attachment activity, which was, at least partly achieved via the PI3K/Akt axis. PPL knockdown is therefore related to reduced cellular movement and attachment activity rather than malignant progression, that is, PPL potentially participates in EMT in pharyngeal cancer cells.

## Methods

### Patients and tissue samples

Patients with pathologically proven hypopharyngeal squamous cell carcinoma (all primary cases) hospitalized at the Department of Otorhinolaryngology at Chiba University Hospital were investigated in this study. Written informed consent was obtained from each patient prior to surgery.

### Cell lines

The D562 pharyngeal cancer cell line was purchased from Human Science Research Resources Bank, Osaka, Japan. TE4, TE9, and TE11 human esophageal cancer cell lines were provided by RIKEN BRC (National Bio-Resource of the MEXT, Tsukuba, Japan) [32,33]. The human esophageal cancer cell lines YES5, TE2, and TTn were kindly provided by Dr. Shimada [34]. All cell lines were cultured at 37°C in a humidified atmosphere containing 5% CO_2 _and maintained in IMDM (Iscove's modified Dulbecco's medium; Gibco BRL, NY, USA) in tissue flasks supplemented with 10% heat-inactivated FBS and penicillin (100 units/mL) as well as streptomycin (0.1 mg/mL).

### Transient siRNA transfection

Double-stranded siRNA oligonucleotides against PPL and firefly luciferase (GL2), the negative control, were purchased from QIAGEN (Hilden, Germany). Cells were grown to 30-40% confluence in 24-well plates, and 20 pmol of siRNA oligonucleotides was transfected using Lipofectamine 2000 reagent (Invitrogen, Carlsbad, CA, USA). Cells were processed for each assay, and whole-cell proteins were extracted 48-72 h after transfection. Knockdown efficiency of PPL by siRNA was assessed by immunoblotting.

### Immunoblotting

Cells were dissolved in lysis buffer (7 M urea, 2 M thiourea, 2% 3-[(3-cholamidopropyl)dimethylammonio-]1-propanesulfate, 0.1 M dithiothreitol, 2% IPG buffer (GE Healthcare, Buckinghampshire, UK), and 40 mM Tris) using a Polytron homogenizer (Kinematica, Switzerland) following centrifugation (100,000 × *g*) for 1 h at 4°C. The amount of protein in the supernatant was measured by protein assay (Bio-Rad, Hercules, CA, USA). The proteins were separated by electrophoresis on 7.5% polyacrylamide gels and transferred to a polyvinylidene fluoride membrane (Millipore, Bedford, MA, USA) in a tank transfer apparatus (Bio-Rad, Hercules, CA, USA). The membrane was blocked with 0.5% skim milk in phosphate-buffered saline (PBS) or 0.5% bovine serum albumin (BSA) in tris-buffered saline with Tween 20 (TBST) for 1 h, and probed with a primary antibody diluted in blocking buffer. Anti-PPL (Betyl Laboratory Ltd., Montgomery, TX, USA), anti-phospho-AKT (Ser473 or Thr308), anti-phospho-MAPK (Cell Signaling Technology, Beverly, MA, USA), and anti-β-actin (Santa Cruz, CA, USA) were used as primary antibodies. Donkey anti-rabbit IgG horseradish peroxidase (HRP) conjugate (Amersham Pharmacia Biotech, Piscataway, NJ, USA) diluted 1:3,000 and rabbit anti-goat IgG HRP (Cappel, West Chester, PA, USA) diluted 1:500 were used as secondary antibodies. Antigens on the membrane were detected by ECL™ detection reagents (GE Healthcare, Buckinghamshire, UK).

### Proliferation assay

Five thousand cells were seeded in 24-well Falcon 3072 plates (Becton Dickinson, Lincoln Park, NJ, USA) at 37°C and 5% CO_2_. After various periods, cell number was established by counting with a phase-contrast Leitz microscope (MD, USA).

### Flow cytometry

For cell cycle analysis, 1 × 10^6 ^cells were seeded in 10-cm-diameter culture dishes. Cells were trypsinized, washed with ice-cold PBS, fixed in 70% ethanol, and stored at -20°C. The cells were then treated with RNase (0.2 mg/mL) for 0.5 h at 37°C and stained with propidium iodide at 50 μg/mL. The stained cells were analyzed in a FACSCalibur cytometer (Becton Dickinson, Lincoln Park, NJ, USA), and the results were analyzed with FlowJo software (Tree Star Inc., San Carlos, CA, USA).

### BrdU incorporation assay

The BrdU incorporation assay was carried out as described previously, using the BrDu flow kit (Becton Dickinson, Lincoln Park, NJ, USA) according to the manufacturer's instructions [35]. In brief, 1-2 × 10^6 ^cells were labeled with 10 μM BrdU for 1 h, harvested, fixed, and treated with DNAase. Cells were stained with anti-BrdU FITC-coupled antibody and 7-AAD for DNA. Samples were analyzed in a FACSCalibur cytometer (Becton Dickinson, Lincoln Park, NJ, USA) using the FlowJo program (Ashland, OR, USA). BrdU signals were recorded on a logarithmic scale in the FL1 channel, and DNA was recorded on a linear scale in the FL3 channel. Single cell events were distinguished from cell aggregates on a FL3-A versus FL3-W plot of 7-AAD fluorescence.

### Wound-healing assay

Cells were transfected with PPL siRNA or pretreated for 2 h with PI3K inhibitor, LY294002 (Wako, Osaka, Japan). Then, cells were grown to confluence in 6-well plates, and monolayer cells were scraped using a micropipette (yellow) tip. After washing with PBS, serum-free medium was added to prevent cell proliferation. Photographs of the wounded area were taken immediately after the scratch was made. Six and 24 h after scraping, cell movement into the wounded area was monitored by time-lapse microscopy.

### Adhesion assay

Adhesion of PC-3 cells was measured as described previously, with some modifications [36,37]. In brief, each well of a 24-well plate was coated with 200 μg/ml Matrigel (Becton Dickinson, Lincoln Park, NJ, USA). In each well, 2 × 10^4 ^cells were added in 0.5 mL serum-free media supplemented with 0.1% BSA. The plates were incubated at 37°C and adhesion was determined at 0.5 h. The plates were fixed with methanol and stained with Diff-Quik (Sysmex Corp., Kobe, Japan). The number of cells adhered to the Matrigel was counted at 4 random fields per well in duplicate at a magnification of ×400.

### Time-lapse microscopy

Time-lapse recordings of cells were made with an Axiovert 200 M SP LSM 510 META confocal laser scanning microscope (Carl Zeiss Inc., Oberkechen, Germany) equipped with an AxioCam charge-coupled device (CCD) camera (Zeiss, Germany) and AxioVision software. Cells were maintained in an environmental chamber at 37°C with 5% CO_2 _during the analysis. Images were taken by CCD camera through a laser scanning microscope every 5 min. Cell tracking was performed using AxioVision software. Images were imported into Adobe Photoshop and prepared as pictures.

### Immunohistochemical staining

Immunohistochemical procedures for PPL expression have been described previously [38]. In brief, air-dried 4-micron cryostat sections were fixed in cold acetone for 20 min and washed with PBS for 5 min. The sections were then incubated with anti-PPL antibody overnight at 4°C. After washing with PBS, the sections were incubated with biotinylated anti-rabbit IgG as a secondary antibody. The sections were then incubated with HRP-conjugated streptavidin-biotin complex for 0.5 h. Peroxidase activity was visualized by DAB solution (20 mg of 3,3'-diaminobenzidine, 65 mg of sodium azide, and 10 mL of 30% hydrogen peroxide in 100 mL of 0.05 M Tris buffer at pH 7.6). Hematoxylin was used for nuclear counterstaining.

### Statistical Analysis

The abovementioned experiments were repeated at least 3 times. Student's t-test was used for statistical analysis.

## Abbreviations

PPL: periplakin; siRNAs: short interfering RNAs; PKB: protein kinase B; EMT: epithelial-mesenchymal transition; ECM: extracellular matrix; PI3K: phosphatidylinositol 3' kinase.

## Competing interests

The authors declare that they have no competing interests.

## Authors' contributions

YT performed all experiments. YT and KM designed the experiment and prepared the manuscript. TT, KK, NT, HS, FN and YO contributed scientific and financial support. FN is responsible for the entire study. All the authors have read and approved the final manuscript.

## Supplementary Material

Additional file 1 **Adhesion assay of D562 cells**. Click here for file

Additional file 2 **siRNA against PPL reduces adhesion to ECM in D562 cells**. D562 cells were transfected with either control siRNA (Additional file 1) or PPL siRNA (Additional file 2). The cells were grown to confluence in glass-bottomed dishes and EDTA (2.5 mM final concentration) was added. Images were analyzed by time-lapse confocal laser scanning microscopy using a laser-scanning confocal microscope (LSM 510 META; Carl Zeiss Inc). Frames were taken every 5 min for 6 h. Click here for file

Additional file 3 **Migration of D562 cells**. Click here for file

Additional file 4 **siRNA against PPL reduces cell migration in D562 cells**. D562 cells were transfected with either control siRNA (Additional file 3) or PPL siRNA (Additional file 4). After 24 h, cells were seeded on glass-bottomed dishes and incubated for another 24 h. Images were analyzed by time-lapse confocal laser scanning microscopy using a laser-scanning confocal microscope. Frames were taken every 5 min for 24 h. Click here for file

Additional file 5 **pAktSer473 phosphorylation affects cellular movement in D562 cells**. (A) (B) 5 × 10^5 ^YES5 cells (low PPL expression) and D562 cells (high PPL expression) were injected beneath right thigh of 8-week male nude mice and monitored subsequent tumor growth. No tumor growth was observed in YES5 mice, while some tumors were observed in D562 mice. (C) LY294002, a PI3K inhibitor, suppressed pAktSer473 phosphorylation but did not alter PPL expressio in D562 cells. (D) LY294002 suppressed D562 cell migration revealed by wound-healing assay in D562 cells. (E) The ratio of width at 6 hrs/0 hr (%) indicated that LY294002 suppressed wound-healing in D562 cells. Click here for file
