# Supplementary figures and images for: Adhesion molecule periplakin is involved in cellular movement and attachment in pharyngeal squamous cancer cells
Source: BMC Cell Biol. 2011 Sep 27;12:41. doi: 10.1186/1471-2121-12-41 (PMC3195110; doi:10.1186/1471-2121-12-41)

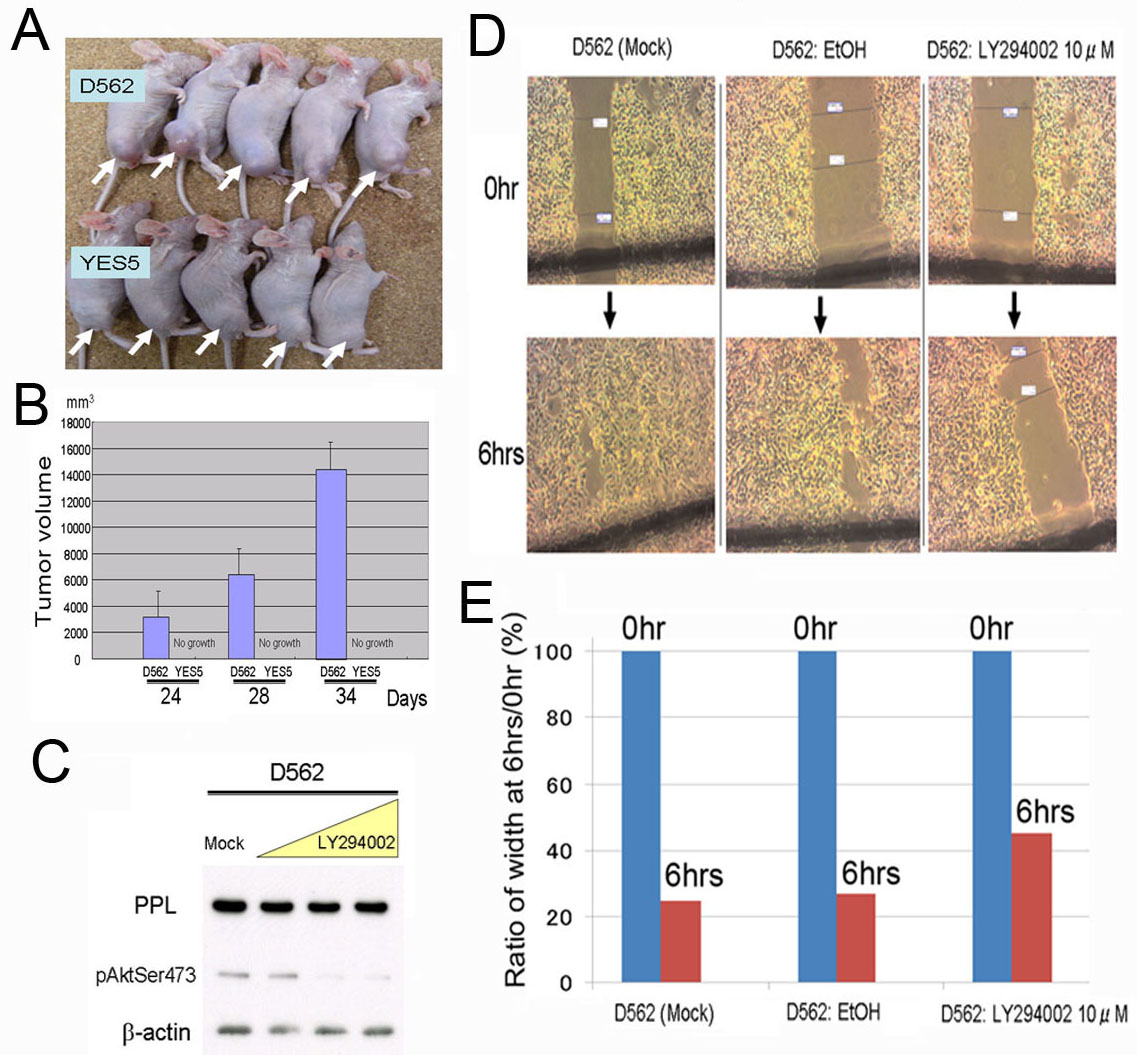

Supplement: Additional file 5 — pAktSer473 phosphorylation affects cellular movement in D562 cells. (A) (B) 5 × 10^5^ YES5 cells (low PPL expression) and D562 cells (high PPL expression) were injected beneath right thigh of 8-week male nude mice and monitored subsequent tumor growth. No tumor growth was observed in YES5 mice, while some tumors were observed in D562 mice. (C) LY294002, a PI3K inhibitor, suppressed pAktSer473 phosphorylation but did not alter PPL expressio in D562 cells. (D) LY294002 suppressed D562 cell migration revealed by wound-healing assay in D562 cells. (E) The ratio of width at 6 hrs/0 hr (%) indicated that LY294002 suppressed wound-healing in D562 cells. [file 1471-2121-12-41-S5.JPEG]
